# Prevalence and Associated Risk Factors of Urinary Schistosomiasis in Al Managil City, Gezira State, Sudan: A Cross‐Sectional Study

**DOI:** 10.1002/hsr2.72575

**Published:** 2026-05-29

**Authors:** Mohamed Ahmed Salah Mohamed Ahmed, Hajrhma Ismael Hajrhma Mohammedahmed, Abdulaziz Gasim Alsid Ahmed, Khadega Suleiman Mohammed Zarroug, Amgad Albashir Khalid Albashir, Mohamed Hassan Ahmed Kebayer, Abobaker Mohamed Al Amin Al Basher, Tafawl Ibrahim Karrar

**Affiliations:** ^1^ Department of Community Medicine, Faculty of Medicine and Health Sciences University of Kassala Kassala Sudan; ^2^ Medical Laboratory Specialists in Medical Clinics Al Managil Gezira State Sudan; ^3^ Department of Microbiology and Parasitology, Faculty of Medicine and Health Sciences University of Kassala Kassala Sudan; ^4^ Microbiology Specialists in Medical Clinics Al Managil Gezira State Sudan; ^5^ Department of Parasitology and Medical Entomology, Faculty of Medical Laboratory Sciences University of Kassala Kassala Sudan; ^6^ Department of Clinical Chemistry, Faculty of Medical Laboratory Sciences University of Kassala Kassala Sudan

**Keywords:** Al Managil, Gezira state, risk factors, urinary schistosomiasis

## Abstract

**Background and Aims:**

Urinary schistosomiasis has remained one of the most important public health issues in Sudan, especially in regions like Gezira State, which relies on irrigation schemes. Despite control activities, morbidity from schistosomiasis in these regions remains a challenge, therefore updated prevalence data are important for proper planning of control strategies. The purpose of this study was to investigate the prevalence and associated socio‐demographic and behavioral risk factors of urinary schistosomiasis among individuals attending healthcare facilities in Al Managil, Gezira State, Sudan.

**Methods:**

A hospital‐based cross‐sectional study was conducted in Al‐Managil city, Gezira State, from August 2022 to June 2023. A total of 212 participants were selected using systematic random sampling. Urine examination was performed microscopically for the presence of eggs of *Schistosoma haematobium*. Data on socio‐demographic, environmental, and behavioral factors were obtained using a structured questionnaire. Data were analyzed using SPSS version 23. The Chi‐square test was used to assess associations between urinary schistosomiasis and its risk factors. *p*‐value < 0.05 was considered to be statistically significant.

**Results:**

The prevalence of urinary schistosomiasis was 29.7% (63/212). The prevalence was significantly higher among males and rural residents, individuals with low educational level, and farmers (*p* < 0.05). Proximity to water source, use of non‐sanitary toilets, bathing, swimming in water, and water contact were also significantly associated with urinary schistosomiasis (*p* < 0.05). Significant associations between urinary schistosomiasis and its symptoms, like hematuria, dysuria, frequency, itching, and urticaria, were also found. Urinary schistosomiasis was not significantly associated with other factors like age, fever, and weight loss.

**Conclusion:**

Urinary schistosomiasis transmission still persists in Al Managil city despite the ongoing control efforts. Male gender, rural residence, poor sanitary conditions, and unsafe behavior concerning water sources have shown to be the significant risk factors for the transmission of the disease. Health education, sanitary conditions, and unsafe water sources need to be addressed to reinforce the continued efforts for the control of the disease.

## 1 Introduction

Urinary schistosomiasis, primarily caused by the trematode parasite *Schistosoma haematobium*, remains a significant public health challenge in many regions, particularly in sub‐Saharan Africa [1]. In Sudan, the prevalence of this disease is notably high, especially in areas with extensive irrigation systems, such as the Gezira State, where the interaction between human activities and environmental factors exacerbates the transmission [2]. The disease is characterized by various clinical manifestations, including hematuria and urinary tract complications, which can lead to severe morbidity if left untreated [3]. The socio‐economic implications of urinary schistosomiasis are profound, affecting productivity and healthcare costs, thereby necessitating urgent public health interventions [4, 5].

The prevalence of urinary schistosomiasis in Sudan has been documented in various studies, highlighting alarming rates in different areas [6, 7]. These figures show the widespread nature of the disease, particularly in areas with significant exposure to contaminated water sources, such as irrigation canals and rivers [8]. In A study done among Gezira State residents, *S. haematobium* pooled prevalence was 41% (95% CI: 26.72, 55.29), which played a significant role in the overall urinary schistosomiasis prevalence in Sudan [9].

Several risk factors have been associated with urinary schistosomiasis, including socio‐demographic variables and occupational exposure. Research has shown that children, particularly those whose fathers are farmers, are at a higher risk of infection due to their frequent contact with contaminated water during agricultural activities [10]. Additionally, socio‐economic factors such as inadequate sanitation and hygiene practices significantly contribute to the transmission of schistosomiasis [11]. The importance of water‐related activities, including swimming and washing, has been identified as a key risk factor in multiple studies, suggesting that behavioral habits play a significant role in determining infection rates [12, 13].

Control measures have been implemented in Sudan, including mass drug administration programs aimed at reducing the prevalence of schistosomiasis [14]. These measures along with snail management and enhanced access to safe water have successfully decreased schistosomiasis prevalence among school‐aged children from more than 70% to below 10% in just 3 years, offering a viable model for Al Managil [15].

However, the effectiveness of these interventions varies, and continuous monitoring is essential to adapt strategies to local contexts [16].

Although several studies have been done in the Sudan on the epidemiology of urinary schistosomiasis in the country in general and in Gezira State in particular, there has been a lack of epidemiological studies in the last period which have relied on a population attending a medical facility in assessing the burden of the disease in the context of continuing control efforts against the parasite in the country in general and in Al Managil in particular. In the case of Al Managil, the studies have been done on a population attending a school or a population within the local population before or at the early stage of the control efforts against the parasite.

This study aims to investigate the prevalence and associated risk factors of urinary schistosomiasis in Al Managil city, Gezira State, Sudan, contributing to the understanding of this neglected tropical disease in a critical endemic region.

## 2 Materials and Methods

### 2.1 Study Design and Study Area

A cross‐sectional, hospital‐based study was conducted in Al Managil city, Gezira State, from August 2022 to June 2023. The design of a cross‐sectional study was chosen in line with the standard epidemiological methodology of estimating the prevalence of disease and associated factors in defined populations. Such a study design was adopted from the works of Gordis [17] and Levin et al [18].

This manuscript was prepared in accordance with Strengthening the Reporting of Observational Studies in Epidemiology guidelines for cross‐sectional studies (STROBE).

Al Managil City is located approximately 153 km south of Khartoum. It can be found at around 14.25° North latitude and 33° West longitude. Its position can be identified on a map of Sudan in the eastern‐central part of the nation [19].

**FIGURE 1:**
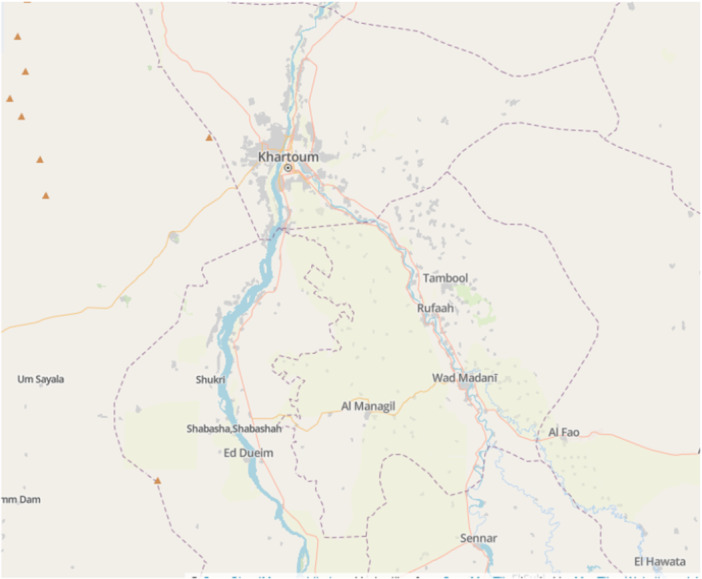
Location of Al Managil city relative to Khartoum capital city.

The geographical location of the study area is shown in Figure 1 below, which illustrates the location of Al Managil city relative to the capital city Khartoum [20].

It had a population of 99,775 as of the 2008 census. Estimates indicate that the population has risen to 143,000, while the 2012 estimates from World Gazetteer indicate that the population stands at 137,739. Based on accurate population data, Al Managil City is the largest city in Al Gezira Province, succeeding Wad Medani. It is also the capital of Al‐Managil district, which is a subdivision in Sudan's state. It is situated in the center of an irrigated region used for agriculture, such as cotton, wheat, and sorghum, and is a retail and service center [21].

Two health centers were selected randomly from the total list of health facilities in the city through simple random sampling method. The total list of health facilities in Al Managil city includes the following centers: Al Shaiq Health Center, Wladalbor Health Center, Al Nasr Health Center, Um Selma Health Center, Al Bir Health Center, Al Ansari Health Center, Al Amal Health Center, Al Belsam Health Center, and El Managil Hospital (*n* = 9).

### 2.2 Study Participants, Sample Size, and Sampling Technique

The study enrolled a total of 212 participants who sought medical care at the selected Al Nasr Health Center and Al Shaiq Health Center in El Managil city. The sample size was determined using the formula (*n* = *z* ^2^ × *p* × *q*/*d* ^2^) [22], where a prevalence rate of 20% was obtained from a previous study [23]. To calculate the sample size, a significance level (*z*) of 1.96 at a 95% confidence interval and a margin of error (*d*) of 5% were employed. Initially, the calculated sample size was 246. Due to the feasibility of recruitment within the study period and the strict criteria for inclusion, a total of 212 participants meeting all the criteria for the study were included.

Despite substituting for any refusals and ineligible individuals by randomly selecting new respondents daily, the total number of refusals and ineligible individuals over the duration of the study led to an attained sample size of 212 (86.2% of the target 246).

Before recruiting participants, a proportional distribution was determined from the average daily number of outpatients per clinic to facilitate recruitment and reduce selection bias among clinics Table 1.

**TABLE 1:** Proportion distribution of the health facilities.

Facility	Target (n = 246)	Proportion	Daily Outpatients
Al Shaiq Health Center	134	54.50%	30
Al Nasr Health Center	112	45.50%	25
TOTAL	246	100%	55

All eligible patients who attended either health center were recruited for participation.

On a daily basis, the research team screened all outpatient registration files at both health centers for patients satisfying the general eligibility requirements.

Every day, a patient was selected randomly. If this patient declined participation in the study or was ineligible, then another patient was selected from the remaining patients randomly this resulted in a rate of approximately 1 patient per facility per day.

The effective rate accounted for days with zero recruitment due to closures or lack of eligible patients.

In addition, a post hoc power analysis revealed that the actual recruited sample (i.e., 212 participants) still had enough statistical power (> 80%) to detect the outcome variable (prevalence of urinary schistosomiasis) at 5% level of significance.

The obtained sample size was 54.7% and 45.3% from Al Shaiq and Al Nasr Health Centers, respectively, which is relatively proportional compared to 54.5% and 45.5%.

### 2.3 Inclusion Criteria

1. Persons of any gender aged greater than 1 year attending specific health facilities during the study period.
2. Residents of Al‐Managil locality for at least 6 months.
3. Persons willing to submit a urine sample and answer the questionnaire.
4. The participants who were at least 18 years old and gave their written informed consent to participate. For minors below 18 years old, parental or legal guardianship consent, along with minor assent.

### 2.4 Exclusion Criteria

1. Persons who received praziquantel treatment in the past 3 months.
2. Persons with severe medical conditions that do not allow an interview or urine sample submission.
3. Subjects who had incomplete or faulty questionnaires, considered to have questionnaires that had large gaps of missing information (e.g., socio‐demographic and exposure information not available), as well as subjects whose urine specimens were insufficiently collected or processed for laboratory testing.

## 3 Data Collection Procedures

### 3.1 Personnel, Qualifications, and Training for Recruitment

The data collection exercise was carried out by a team consisting of medical laboratory specialists who were trained in the field of medical laboratory science and final year medical and medical laboratory sciences students from the University of Kassala. The team was supervised by senior researchers and a parasitologist. Prior to conducting the data collection process, all team members had been exposed to the basics of epidemiological and clinical data gathering exercises.

Prior to the start of data gathering, all team members received comprehensive training sessions from the principal investigators. This included an orientation on the objectives of the study, participant selection criteria, recruitment procedure, ethics (informed consent and confidentiality), questionnaire administration, sample collection, and packaging. Practical demonstrations were also conducted in order to ensure consistency in interview and laboratory procedures. A pilot test was also conducted to ensure consistent application of study procedures among all participants.

### 3.2 Description of the Lottery Sampling Technique

To ensure that only randomly selected individuals participated in the study, the researchers used the lottery sampling technique. Every day, a list of all eligible participants who attended the health facilities' outpatient departments was extracted based on the set inclusion and exclusion criteria.

All the eligible participants were provided with sequential numbers. These numbers were then put down on identical papers that were subsequently folded for concealment purposes and placed in a bowl. The numbers were randomly selected by a researcher who had no role in assessing the patients. The participant with the selected number was asked to join the study.

In a situation where the individual rejected to take part in the study or failed to satisfy the criteria after the initial selection, the process was repeated until one participant was recruited each day in the facility under study.

### 3.3 Laboratory Diagnosis of *Schistosoma haematobium*

1. Collection of urine samples and the urine samples were gathered between 10:00 a.m. and 2:00 p.m., as this is the ideal time for detecting *S. haematobium* eggs, with excretion levels peaking during this window.
2. Each participant received a sterile, pre‐labeled urine collection container that had a unique identification code. This code corresponded with the participant's questionnaire ID to ensure accurate linkage and avoid mix‐ups.
3. Once collected, the urine samples were taken to the laboratory and analyzed within 2–4 h. A 10 mL portion of each sample was centrifuged at 3000 rpm for 5 min, and the resulting sediment was examined under a microscope using ×10 and ×40 objectives. The presence of *S. haematobium* eggs was verified by their distinctive oval shape featuring a terminal spine [16].
4. To ensure quality control, all slides were examined by qualified laboratory personnel, and a second examination of a sample of slides was carried out by another parasitology Lab specialist to eliminate observer bias.

### 3.4 Diagnostic Limitations

The diagnosis of urinary schistosomiasis infection in this study was done through the microscopy of a single urine sample, which may not give the accurate prevalence of the infection in the community due to the daily fluctuation in the excretion of eggs in the urine of infected persons. More sensitive methods of diagnosing the infection, such as urine filtration, were not used due to the limitations of the study.

### 3.5 Questionnaire‐Based Risk Factor Assessment

A structured and validated questionnaire was used, which was administered through face‐to‐face interviews with the respondents using data collectors. The questionnaire was intended to measure socio‐demographic factors, environmental exposures, behavioral factors, and clinical symptoms that may be associated with urinary schistosomiasis.

Variables of interest were the following: age, gender, educational level, occupation, place of residence, proximity to water bodies, sanitation facilities, source of drinking water, and water contact activities like swimming, bathing, fishing, laundry, and self‐reported urinary symptoms like hematuria, dysuria, and frequency of urination. These were the main variables of interest to this study; however, the questionnaire also contained additional questions on environmental exposures and health history.

The symptoms presented were based on the self‐report by the participant.

For the purpose of consistency and reproducibility of results, certain variables were defined. For instance, the proximity of the household to water bodies was defined as the self‐reported distance of the participant's household from irrigation canals, rivers, or other open water sources and was categorized into either near or far from the household residence, where near was defined as ≤ 1 km and far was defined as > 1 km from the household residence.

The questionnaire was developed after conducting an extensive review of the relevant literature regarding the risk factors for urinary schistosomiasis. The questionnaire was written in English and then translated into Arabic to make it easier to comprehend for the respondents. Before the actual data collection process, the questionnaire was pre‐tested among 10 respondents who did not form part of the final study population to check the clarity, relevance, and understanding of the questions asked. Based on the feedback received during the pilot study, the questionnaire was modified accordingly.

### 3.6 Safety, Privacy, and Confidentiality of Participants

Each participant was given a unique identification number that linked the information obtained from the questionnaire and the urine sample without obtaining personal information such as names and addresses.

The interviews were carried out in a private area within the healthcare facilities to ensure confidentiality of the information obtained from each participant. The information obtained from each participant was stored securely and only accessible by members of the research team. The participants were also given the freedom of choice and were informed that they could withdraw from the study at any given time without affecting their medical care.

### 3.7 Statistical Analysis

Analyses that assessed the prevalence of urinary schistosomiasis and its associations with socio‐demographic and environmental variables, including sex, age group, residence, education, occupation, water contact, sanitation, and bathing habits, had been pre‐specified before data analysis. Analyses that assessed associations between urinary schistosomiasis and clinical symptoms, as well as subgroup analyses conducted within demographic categories, were regarded as exploratory and interpreted appropriately.

The analysis was done using the Statistical Package for Social Sciences, version 23, provided by IBM Corporation, Armonk, NY, USA. Frequency distributions and percentages were used for categorical data. The prevalence estimate of urinary schistosomiasis was also computed with its confidence intervals set for 95%. The association between urinary schistosomiasis and any other categorical data was also carried out using the test for independent categories *χ* ^2^. Tests carried out were all two‐tailed, and alpha was set for all tests at 0.05. Results obtained by this analysis were presented as tables.

A *p*‐value indicates the probability of observing the data under the null hypothesis; CI stands for confidence interval; and *χ* ^2^ is a test statistic under the Chi‐square distribution.

### 3.8 Ethical Approval

Ethical clearance: The study received ethical clearance from the Ethical Committee of the University of Kassala, Sudan [Reference Number: [MLS‐ERC‐2022‐010]; Date: July 15, 2022].

Informed Consent/Assent: Before enrollment into the study, all subjects or parents/guardians were fully informed about the purpose of the study, procedures involved possible dangers and benefits, right to withdraw, and protection of their privacy.

Adults (>/=18 years old): Informed consent was obtained from each subject personally upon confirmation of their understanding of the study.

Minors (< 18 years old): Informed consent was obtained from parents/legal guardians. Moreover, age‐appropriate assent was obtained from all minor participants.

The reporting and ethical process will enable proper understanding, replicability, and consideration of proper studies in relation to eventual reviews.

## 4 Results

The socio‐demographic characteristics of the study participants revealed a predominantly male 133 (62.7%) compared to females 79 (37.3%). Most participants reside in urban areas 129 (60.8%), while 83 (39.2%) are from rural settings. Age distribution shows a significant youth presence, with 81 (38.2%) aged 16–30 and 58 (27.4%) aged 1–15. Older age groups are less represented: 34 (16.0%) aged 31–45, 26 (12.3%) aged 46–60, and 13 (6.1%) over 60. Regarding education, 63 (29.7%) have secondary education, while 30 (14.2%) are illiterate, and only 23 (10.8%) hold a university degree. Occupationally, 159 (75.0%) fall under the “Other” category, with farmers at 41 (19.34%), employees at 9 (4.25%), and fishermen at 3 (1.41%) (Table 2).

**TABLE 2:** Socio‐demographic characteristics of study participants.

Characteristics	Frequency	Percent (%)
Gender		
Male	133	62.7%
Female	79	37.3%
Residency		
Urban	129	60.8%
Rural	83	39.2%
Age groups		
1–15	58	27.4%
16–30	81	38.2%
31–45	34	16.0%
46–60	26	12.3%
> 60	13	6.1%
Educational level		
Illiterate	30	14.2%
Primary school	51	24.1%
Intermediate school	45	21.2%
Secondary school	63	29.7%
University	23	10.8%
Occupation		
Farmer	41	19.34%
Employee	9	4.25%
Fisherman	3	1.41%
Other	159	75.0%

The results showed that out of a total of 212 participants, 63 (29.7%) tested positive for urinary schistosomiasis, while the majority of the participants, 149 (70.3%) tested negative (Table 3).

**TABLE 3:** Prevalence of urinary schistosomiasis among study participants.

Characteristics	Frequency	Percent (%)	95% CI
Urinary schistosomiasis			
Positive	63	29.7%	23.7–36.4
Negative	149	70.3%	63.6–76.3

Table 4 presents a comprehensive analysis of socio‐demographic characteristics and risk factors associated with urinary schistosomiasis. The results showed a significant gender disparity, with males exhibiting a higher prevalence (27.8%) compared to females (1.9%), with a statistically significant *p* < 0.001. Residency also plays a crucial role, as rural inhabitants showed a higher infection rate (22.2%) compared to their urban counterparts (7.5%), again with a *p* < 0.001. In terms of age, there are significant positivity rates of 10.4% and 11.8% in the 1–15 and 16–30 age groups, respectively, although there are low positivity rates in older age groups; however, in terms of association, the *p*‐value in relation to age groups was found to be 0.23, indicating that there was no significant association. The age groups considered, that is, 1–15, 16–30, 31–45, 46–60, and over 60, are logical as they conform to set. Educational level appeared to correlate with infection rates, particularly at the primary school level (9.9% positive), with a *p* = 0.010 suggesting some level of significance. Occupationally, other and farmers showed a higher prevalence, 15.6% and 13.7%, respectively, compared to employees and fishermen with a *p* < 0.001. Proximity to water sources is markedly significant, with those living near water showing a higher positivity rate (25.5%) vs. those living farther away (4.2%), *p* < 0.001. The use of sanitary toilets is related to infection rates, with 18.4% of those using non‐sanitary toilets testing positive compared to 11.3% using sanitary toilets, also significant at *p* < 0.001. Bathing habits reveal that home bathing is associated with a higher positivity rate (18.4%) compared to canal bathing (7.1%), with a *p* < 0.001. Contact with water and swimming habits further elucidate risk factors, with individuals who have had water contact showing a higher positivity rate (24.5%) and those who swim also reflecting significant rates (17.9%), both with *p* < 0.001.

**TABLE 4:** Associated risk factors of urinary schistosomiasis in Managil City, Gezira State, Sudan.

Characteristics and risk factors	Positive	95% CI	Negative	95% CI	*X* ^2^	*p* value
Gender					36.645	< 0.001
Male	59 (27.8%)	21.9–34.4	74 (34.9%)	28.5–41.7		
Female	4 (1.9%)	0.5–4.8	75 (35.4%)	29–42.2		
Residency					47.291	< 0.001
Urban	16 (7.5%)	4.4–12	113 (53.3%)	46.3–60.2		
Rural	47 (22.2%)	16.8–28.4	36 (17.0%)	12.2–22.7		
Age groups					5.664	0.23
1–15	22 (10.4%)	6.6–15.3	36 (17.0%)	12.2–22.7		
16–30	25 (11.8%)	7.8–16.9	56 (26.4%)	20.6–32.9		
31–45	8 (3.8%)	1.6–7.3	26 (12.3%)	8.2–17.5		
46–60	7 (3.3%)	1.3–6.7	19 (9.0%)	5.5–13.6		
> 60	1 (0.5%)	0.0–2.6	12 (5.7%)	3.0–9.7		
Educational level					13.266	0.010
Illiterate	13 (6.1%)	3.3–10.3	17 (8.0%)	4.7–12.5		
Primary school	21 (9.9%)	6.2–14.7	30 (14.2%)	9.8–19.6		
Intermediate school	14 (6.6%)	3.7–10.8	31 (14.6%)	10.2–20.1		
Secondary school	13 (6.1%)	3.3–10.3	50 (23.6%)	18.0–29.9		
University	2 (0.9%)	0.1–3.4	21 (9.9%)	6.2–14.7		
Occupation					42.961	< 0.001
Farmer	29 (13.7%)	9.4–19.1	12 (5.7%)	3.0–9.7		
Employee*	0 (0.0%)	0.0–1.7	9 (4.2%)	2.0–7.9		
Fisherman	1 (0.5%)	0.0–2.6	2 (0.9%)	0.1–3.4		
Other	33 (15.6%)	11.0–21.2	126 (59.4%)	52.5–66.1		
Distance from water source**					57.668	< 0.001
Near	54 (25.5%)	19.8–31.9	43 (20.3%)	15.1–26.3		
Far	9 (4.2%)	2.0–7.9	106 (50.0%)	43.1–56.9		
Using toilet					18.863	< 0.001
Sanitary toilet	24 (11.3%)	7.4–16.4	18 (8.5%)	5.1–13.1		
None sanitary toilet	39 (18.4%)	13.4–24.3	131 (61.8%)	54.9–68.4		
Bathing habit					39.717	< 0.001
Home	39 (18.4%)	13.4–24.3	142 (67.0%)	60.2–73.3		
Canal	15 (7.1%)	4.0–11.4	5 (2.4%)	0.8–5.4		
River	0 (0.0%)	0.0–1.7	0 (0.0%)	0.0–1.7		
Both home and canal	9 (4.2%)	2.0–7.9	2 (0.9%)	0.1–3.4		
Contact of water					82.790	< 0.001
Yes	52 (24.5%)	18.9–30.9	25 (11.8%)	7.8–16.9		
No	11 (5.2%)	2.6–9.1	124 (58.5%)	51.5–65.2		
Swimming					60.770	< 0.001
Yes	38 (17.9%)	13.0–23.8	15 (7.1%)	4.0–11.4		
No	24 (11.3%)	7.4–16.4	135 (63.7%)	56.8–70.2		

*Note:* Chi‐square test, *p*‐value < 0.05 considered statistically significant.

* Employee considers all indoor jobs.

** Distance from water source‐near was defined as ≤ 1 km and far was defined as > 1 km from the household residence.

The relationship between urinary schistosomiasis and symptoms showed significant associations were found for skin itching, skin redness, urticaria, hematuria, dysuria, and frequent urination, all with *p*‐values < 0.05. In contrast, fever and weight loss showed no significant correlation (*p*‐values 0.24 and 0.66, respectively) (Table 5).

**TABLE 5:** Relationship between symptoms and urinary schistosomiasis.

Symptom	Positive		Negative		*X* ^2^	*p*‐value
	Yes	No	Yes	No		
Skin itching	8 (3.8%)	55 (25.9%)	3 (1.4%)	146 (68.9%)	10.276	0.003
Skin redness	9 (4.2%)	1 (0.5%)	54 (25.5%)	148 (69.8%)	18.261	< 0.001
Urticaria	31 (14.6%)	7 (3.3%)	32 (15.1%)	142 (67.0%)	59.623	< 0.001
Hematuria	60 (28.3%)	33 (15.6%)	3 (1.4%)	116 (54.7%)	96.063	< 0.001
Dysuria	5 (2.36%)	1 (0.47%)	58 (27.36%)	148 (69.81%)	8.499	0.009
Frequent urination	50 (23.6%)	20 (9.4%)	13 (6.1%)	129 (60.8%)	87.057	< 0.001
Fever	4 (1.9%)	4 (1.9%)	59 (27.8%)	145 (68.4%)	1.638	0.24
Weight loss	1 (0.47%)	2 (0.94%)	62 (29.25%)	147 (69.34%)	0.019	0.66

## 5 Discussion

The data obtained reflects the current status of the epidemiological situation of urinary schistosomiasis infection in Al Managil, where years of control program implementation have provided an opportunity to assess progress and factors driving transmission of this infection.

The observed prevalence rate of 29.7% (95% CI: 23.7%–36.4%) implies that there is still considerable public health impact due to urinary schistosomiasis in Al Managil, even though it is considerably less than the 56%–62% previously seen in South Darfur State [6, 7]. The discrepancy may be attributed to differences in preventive chemotherapy access, density of irrigation facilities, and levels of socio‐economic development in these two regions. However, the prevalence in Al Managil is higher compared to other parts of Gezira State despite the implementation of a robust control program [24], implying that the extensive canal system present in the region, alongside constant human contact with water, continues to support the transmission process. The Gezira irrigation scheme is one of the most expansive in Africa, providing an ideal environment for *Bullins* snail intermediate hosts [4, 14].

The higher rates of male infection are consistent with existing epidemiological trends in schistosomiasis [25, 26]. Such gender differences are not purely numerical but stem from deep‐seated occupational and leisure practices. Given Al Managil's rural setting, males are predominantly involved in irrigation agriculture, fishing, and canal occupations involving extended water exposure. Additionally, swimming, which is predominantly a male practice in this society, constitutes an important yet often overlooked transmission route. Males' susceptibility to infection is no greater than that of females; instead, the difference is primarily due to varying exposures [25].

Rural populations are also affected by a greater burden, which is a manifestation of disparities in resource distribution and infrastructural development [27, 28]. In Al Managil, rural households heavily depend on water from the canals for domestic purposes such as washing, bathing, and cooking, since they lack access to potable water through pipelines. Furthermore, there is inadequate sanitation in rural communities, where people practice open defecation around water sources.

This finding requires serious consideration since there is no significant relationship between age and infection (*p* = 0.226). Usually, in the endemic areas, Schistosomiasis follows an age prevalence curve, with a peak among school‐aged children and falling off in adult life due to the development of immunity and reduction in contact with water [29]. This does not happen in Al Managil, probably due to the consistent nature of exposure throughout one's lifetime as everyone is engaged with water activities irrespective of age.

The connection between low education levels and increased chances of infection (*p* = 0.010) mainly occurs via the intermediary effect of health literacy and behavior [30]. People with poor education backgrounds might lack knowledge of how schistosomiasis is transmitted and how to prevent it, resulting in risky behavior in terms of water use.

Occupation turned out to be an important factor as farmers high prevalence rates compared to workers in indoor occupations. Indeed, it is consistent with previous evidence indicating that agricultural occupations are major factors increasing susceptibility to schistosomiasis in irrigated settings [28, 31]. As it can be easily explained, farming implies regular exposure to irrigation water, especially during peak cercarial shedding periods in the warm middle of the day. It is worth noting that occupational groups have been considered quite broadly in this study; it could have been more informative to use narrower occupational categories.

Risk factors of environmental and behavioral origins were found to have the greatest impact on infection. Being close to the water source (< 1 km), not using sanitary toilets, swimming in the canals, and swimming were found to be highly correlated with infection (*p* < 0.001). Together, these results show the various routes of transmission present in Al Managil: infection due to percutaneous exposure while being in contact with water, pollution of the water resources because of indiscriminate defecation, and lack of access to potable domestic water resources leading to dependence on potentially unsafe sources [3, 32]. The occurrence of infection among those who had no record of direct water contact might indicate possible indirect routes of infection through contaminated domestic water resources, vegetation, and fomites [32].

Moreover, the use of sanitary toilets was also correlated with lower infection rates (*p* < 0.001). This finding aligns with observations from Oniya et al., who emphasized the importance of sanitation in controlling Schistosomiasis transmission [33].

Several studies have found associations between urinary schistosomiasis and specific clinical manifestations. Urinary schistosomiasis was significantly associated with dermatological manifestations like itching, redness, and urticaria of the skin (*p* < 0.05), which agrees with Singh et al. [34]. Urinary manifestations like hematuria, dysuria, and frequent urination were significantly associated with urinary schistosomiasis, which agrees with the findings of studies in Angola [35] and Nigeria [28], as well as Markus and Bishop [36].

In contrast, the lack of significant correlation between urinary schistosomiasis and symptoms such as fever and weight loss is noteworthy. While fever is often considered a major symptom in acute Schistosomiasis cases, its absence in chronic infections may reflect the body's adaptation to the persistent presence of the parasite [37]. This aligns with findings from Yusuf et al., who indicated that chronic infections are more associated with symptoms like dysuria and hematuria rather than systemic symptoms such as fever [38]. However, these observations need to be viewed with caution since the present study is cross‐sectional in nature, and no cause–effect relationship can be established.

The results also resonate with the broader epidemiological context of urinary schistosomiasis, where symptoms can vary significantly based on the stage of infection and the host's immune response. For example, ongoing infections can result in more specific symptoms, like those impacting the urinary system, whereas overall symptoms might be less noticeable [39]. This is further supported by the work of Barda et al., who emphasized the importance of understanding the symptomatic profile of urinary schistosomiasis in managing and treating affected populations [40].

### 5.1 Study Limitation

The population attending healthcare services to seek medical attention might be different from the general population in their health status, therefore, the information obtained in this research may not be fully representative of the prevalence of urinary schistosomiasis in the general population of Al‐Managil city.

It should be noted that the symptoms presented in this study were based on the self‐report of the participants. As such, the study should be viewed with some caution, given the fact that the symptoms presented by the participants might be subjected to recall bias or reporting inaccuracies.

## 6 Conclusion

Urinary schistosomiasis transmission still persists in Al Managil city despite the continued efforts for its control. Male gender, rural residence, poor sanitary conditions, and unsafe behavior concerning water sources have shown to be the significant risk factors for the transmission of the disease. Health education, sanitary conditions, and unsafe water sources need to be addressed to reinforce the continued efforts for the control of the disease.

## Author Contributions

**Mohamed Ahmed Salah Mohamed Ahmed:** investigation, conceptualization, resources. **Hajrhma Ismael Hajrhma Mohammedahmed:** conceptualization, writing – original draft, data curation, writing – review and editing, validation. **Abdulaziz Gasim Alsid Ahmed:** investigation, conceptualization, resources. **Khadega Suleiman Mohammed Zarroug:** investigation, conceptualization, methodology, writing – review and editing. **Amgad Albashir Khalid Albashir:** investigation, conceptualization, resources. **Mohamed Hassan Ahmed Kebayer:** conceptualization, writing – original draft, writing – review and editing, methodology, software, formal analysis, data curation, supervision, resources, project administration, validation, visualization. **Abobaker Mohamed Al Amin Al Basher:** conceptualization, investigation, methodology. **Tafawl Ibrahim Karrar:** conceptualization, investigation, methodology, formal analysis.

## Funding

The authors have nothing to report.

## Disclosure

The lead author Mohamed Hassan Ahmed Kebayer affirms that this manuscript is an honest, accurate, and transparent account of the study being reported; that no important aspects of the study have been omitted; and that any discrepancies from the study as planned (and, if relevant, registered) have been explained.

## Conflicts of Interest

The authors declare no conflicts of interest.

## Data Availability

The data that support the findings of this study are available from the corresponding author upon reasonable request.
